# Analysis of dosimetric factors associated with temporal lobe necrosis (TLN) in patients with nasopharyngeal carcinoma (NPC) after intensity modulated radiotherapy

**DOI:** 10.1186/1748-717X-8-17

**Published:** 2013-01-22

**Authors:** Sheng-Fa Su, Shao-Ming Huang, Fei Han, Ying Huang, Chun-Yan Chen, Wei-Wei Xiao, Xue-Ming Sun, Tai-Xiang Lu

**Affiliations:** 1 Department of Oncology, Affiliated Hospital of Guiyang Medical College, Guizhou Cancer Hospital, Guiyang, China; 2 State Key Laboratory of Oncology in Southern China; Department of Radiation Oncology, Cancer Center, Sun Yat-sen University, Guangzhou, China

**Keywords:** Nasopharyngeal carcinoma, Temporal lobe necrosis, Intensity modulated radiotherapy

## Abstract

**Background:**

The radiation tolerance dose-volume in brain remains unclear for nasopharyngeal carcinoma (NPC) patients treated with intensity modulated radiotherapy (IMRT). We performed this study to investigate dosimetric factors associated with temporal lobe necrosis (TLN) in NPC patients treated with IMRT.

**Methods:**

From 2001 to 2008, 870 NPC patients were treated with IMRT. For the whole group, 40 patients have developed MRI-diagnosed TLN, and 219 patients were followed-up more than 60 months. Predictive dosimetric factors for TLN were identified by using univariate and multivariate analysis in these 259 patients.

**Results:**

By univariate analyses, rVX ( percent of temporal lobes receiving ≥ X Gy) and aVX ( absolute volumes of temporal lobes receiving ≥ X Gy, values of X considered were 10, 20, 30, 40, 50, 60, 66 and 70) were all significantly associated with TLN. Multivariate analysis by logistic regression showed that rV40 and aV40 were significant factors for TLN. All dosimetric factors in current serials were highly correlated one another (p < 0.001). The 5-year incidence of TLN for rV40 <10% or aV40 <5 cc is less than 5%. The incidence for rV40 ≥ 15% or aV40c ≥ 10c is increased significantly and more than 20%.

**Conclusions:**

In this study, all dosimetric factors were highly correlated, rV40 and aV40 were independent predictive factors for TLN, IMRT with rV40 <10% or aV40 <5 cc in temporal lobe is relatively safe.

## Background

Nasopharyngeal carcinoma (NPC) is an endemic disease in southern China. Definitive radiotherapy (RT) has been the main treatment modality for non-metastatic NPC. Because it optimizes the radiation deposition in the tumor while sparing the adjacent normal structures, intensity modulated RT (IMRT) has been widely used for NPC and improved clinical outcomes, especially local control. The issue of late complications after IMRT causes increasing concern as the treatment outcome improves. Xerostomia, trismus and temporal lobe necrosis (TLN) are most common long-term side effects for NPC patients after two-dimensional radiation therapy (2D-RT) [1]. It is reported increasingly that IMRT is able to reduce xerostomia and trismus compared with 2D-RT, and dosimetric factors associated with these two complications have been reported [2,3].

It is unavoidable that the temporal lobes (TLs) will be involved in the radiation fields, no matter using IMRT or 2D-RT. Radiation induced temporal lobe necrosis (TLN) hence becomes a common late damage that adversely affects the quality of life and survival of NPC patients after radiotherapy [4-6]. Clinical, dosimetric, and radiographic correlation of TLN in NPC patients after 2D-RT have been reported in previous investigations [4,7,8]. Lee et al. reported that 64 Gy (at conventional fractionation of 2 Gy daily) would lead to a 5% necrotic rate in TLs at 10 years after 2D-RT in NPC patients [7]. Emami’s original estimate for fractionated partial brain RT suggested 5% risk at 5 years for one-third brain irradiated to 60 Gy [9]. Historic data and literature indicate that the incidence of radiation induced-TLN range from 0% to 18.6% after 2D-RT [4,7,10]. However, published data regarding TLN after IMRT in NPC patients remain limited. The precise mechanism that causes TLN remains unknown, but it is generally believed that TLN is associated with volume and dose of TLs irradiated. To date, the radiation tolerance dose-volume in brain remains unclear, though data for detailed brain dose–volume and outcomes are much-needed [11,12]. Therefore, we performed this study to investigate the relationship between the incidence of TLN and IMRT dosimetric factors. This study aims to improve the understanding of TLN and optimize the IMRT planning for NPC.

## Methods

### Patient selection

This retrospective study was approved by the institutional review board. Between May 2001 and January 2008, 870 non-metastatic NPC patients were treated with IMRT at the Cancer Center of Sun Yat-Sen University. The informed consent for treatment was obtained from all patients. The median duration of follow-up for the entire group of patients was 40 months (range, 6–104 months). After IMRT completion, patients were subsequently followed up monthly in the first 3 months, every three months during the first 3 years, in every 6 months during the next 2 years, and then annually. During follow-up time, MRI examination of head and neck was taken at least once every 6 months or 1 year.

The data from these 870 patients were retrospectively analyzed. The purpose of the current study is to investigate dosimetric factors associated with incidence of TLN at 5 years following IMRT treatment. Thus, patients were included if they were followed-up for ≥5 years or diagnosed as TLN based on MRI findings within 5 years after treatment. Patients were not included in current study if they died or were lost from follow-up within 5 years following IMRT without TLN. Totally, 259 patients were enrolled in this study, the male/female ratio was nearly 3.4:1, and age from 18 to 78 years (median, 43 years). According to the AJCC 2002 staging system, total 17 patients were in stage I, 78 in stage II, 112 in stage III, and 52 in stage IVa-b; based on the T-stage classification, there were 30 of T1, 97 of T2, 83 of T3, and 49 of T4. The dosimetric factors of both normal and injured TLs have been collected from dose–volume histogram (DVH) that was recoverable from institutional archives, and dosimetric facrors associated with TLN are analyzed.

### Treatment methods

#### IMRT planning

The prescribed dose was 68 Gy to the planning target volume (PTV) of nasopharyngeal gross tumor volume (GTVnx), 64 to 66 Gy to the PTV of the gross target volume of positive neck lymph nodes (GTVnd), 60 Gy to the PTV of the first clinical tumor volume (CTV1), and 54 Gy to the PTV of the second CTV (CTV2) in 30 fractions. All patients were treated with one fraction daily over 5 days per week. Organs at risk were also outlined for dose constraint evaluation. Inverse IMRT planning was performed using the Corvus system version 3.0 (Peacock, Nomos, Deer Park, IL, USA), and a MIMiC multileaf collimator (Nomos, Sewickly, PA, USA) was used for planning and treatment. The whole process of IMRT was carried out as described previously by Su et al. and Xiao et al. [13,14].

**Figure 1:**
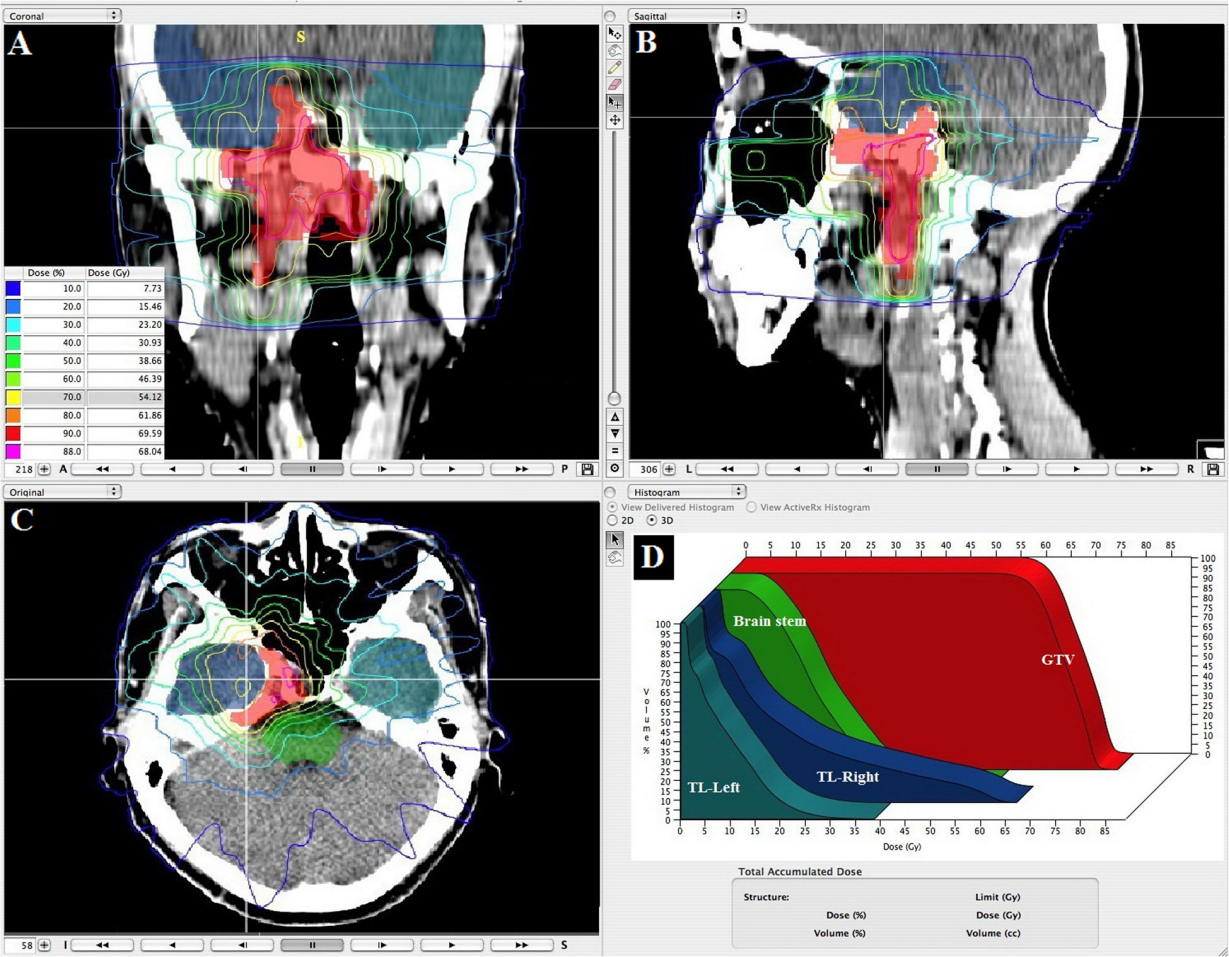
**Isodose curves for an IMRT plan are shown.** The patient had a T4N1M0 cacinoma of the nasopharynx as seen on the coronal (**A**), sagittal (**B**), and axial (**C**) planes. The dose-volume histograms for relevant structures are shown (**D**).

With the help of our radiologist colleague, we develop a institutional protocol to contour the temporal lobes in our cancer centre. That is: the temporal lobes start at region bounded by sphenoid bone, petrous part and squamous part of temporal bone. Then the lobes extend superiorly and posteriorly and end at about the junction of temporal bone and parietal bone. The dose constraints for temporal lobe were to set 5% of the volume (D5) not to exceed 60 Gy. Figure 1 shows a dose distribution of an IMRT plan at the temporal lobe level and the DVH of the temporal lobe for a patient with NPC classified as T4N1M0.

#### Chemotherapy

Our institutional guidelines recommended IMRT alone for patients in stage I to II, and IMRT combined with concurrent chemotherapy for those in Stage III-IVb. Neoadjuvant cisplatin-based chemotherapy was applied in those patients with bulky neck node (>3 cm), and adjuvant chemotherapy was for those patients with residual disease after IMRT. Among 40 patients with TLN, 36 (90%) patients received concurrent chemoradiation.

### Diagnostic criteria for TLN

Tumor recurrence and constitutional symptoms from other diseases and treatments may confound the diagnosis of TLN. Therefore, all TLN were diagnosed based on MRI findings. While T1-weighted and T2-weighted fast spin-echo images in the axial, coronal, and sagittal planes were obtained before injection of the contrast material, T1-weighted and T1-weighted fat-suppressed imaging was performed with the same factors after intravenous administration of gadopentetate dimeglumine (Gd-DTPA). MRI findings were reviewed by two radiologists separately, and any disagreements were resolved by consensus. The diagnostic criteria for TLN were as follows: TLN lesions had contrast enhancement on postcontrast T1-weighted MRI, and T2-weighted images showed corresponding heterogeneous hyperintense necrosis surrounded by homogeneous hyperintense cerebral edema. The recurrence or metastasis of tumor was excluded when determining the site of TLN. MRI findings were reviewed by two radiologists in consensus.

### DVH factors

The following dosimetric factors were generated from the DVH for bilateral temporal lobes respectively: maximal dose (Dmax), mean dose (Dmean), rVX ( percent of temporal lobes receiving ≥ X Gy) and aVX ( absolute volumes of temporal lobes receiving ≥ X Gy), where values of X considered were 10, 20, 30, 40, 50, 60, 66 and 70.

### Statistical analysis

The endpoint for this analysis was occurrence of TLN. The Statistical Package for Social Sciences, version 13.0 (SPSS, Chicago, IL) was used for statistical analysis. The receiver operating characteristic (ROC) curve analysis was applied to evaluate different cut-off point of rVX and aVX in order to find the appropriate value for clinical application. Chi-square test was used in univariate analyses whereas logistic regression was used in multivariate analyses. The correlation between rVX or aVX and the occurrence of TLN was analyzed using linear regression. All statistical tests were two-sided, and *P* < 0.05 was considered as statistically significant.

## Results

### The incidence and latency of TLN

Total 40 of 870 patients (4.60%) have developed MRI-diagnosed TLN, 14 had T_3_ disease, and 26 of T4. Among these 40 patients, 33 (82.5%) have radiation injuries in unilateral temporal lobes; 7 (17.5%) in bilateral temporal lobes. Therefore, total of 47 injured temporal lobes have been detected in these 40 patients.

The median latency for detection of TLN is 30 months (range, 6–56 months), all TLN happened within 5-years after IMRT. Given that the longest latency of TLN in the present study is 56 months, the injury-free TLs of those patients followed-up for ≥60 months are regarded as normal. Based on the anatomy, two sides of temporal lobe in each patient have been analyzed separately. In this study, totally 219 patients without TLN have been followed up ≥5 years and undergone MRI examination at least once over the 60 months after completion of the IMRT to exclude TLN. These 219 patients were enrolled in this study, including 438 normal TLs. Among 40 patients with TLN, 7 had TLN in bilateral temporal lobes, thus 14 injured TLs include in the study. Thirty-three had TLN in unilateral temporal lobes, 16 out of these 33 patients were followed up ≥5 years, 32 TLs (16 normal and 16 injured TLs) of 16 patients were included in the study; 17 out of these 33 patients were followed up < 5 years, including 34 TLs (17 normal and 17 injured TLs), only 17 injured were included in the study. Thus, the actual incidence of TLN at 5 years was analyzed in 259 patients with 454 normal TLs and 47 injured TLs.

### Dosimetric analysis of TLN

Table 1 lists the cut point identified by ROC curve analysis for dosimetric factors associated with TLN, and the incidence of TLN in the resulting subgroups and the results of Chi-square test comparisons of TLN in the subgroups. Significant differences in TLN were found in all subgroups.

**Table 1:** Incidence of TLN in subgroups defined by ROC curve analysis of rVX, aVX, Dmax and Dmean

**Variable**	**Range (median)**	**Group**	**No. of TLs**	**Incidence of TLN**	***χ***^***2***^	***P *value**
rV10	6.8%–100%(37.86%)	≤42%	316	1.9%	53.360	0.000
		>42%	185	22.2%		
aV10	7.32–107.86 cc(29.00 cc)	≤38 cc	358	2.8%		
		>38 cc	143	25.9%	64.033	0.000
rV20	4.16%–100%(21.12%)	≤21%	272	0.7%	52.326	0.000
		>21%	229	19.7%		
aV20	2.93–107.86 cc(16.02 cc)	≤17 cc	294	0.7%	63.368	0.000
		>17 cc	207	21.7%		
rV30	1.80%–99.99%(12.67%)	≤20%	364	2.7%		
		>20%	137	27%	68.911	0.000
aV30	1.10–107.85 cc(9.39 cc)	≤15 cc	358	2.2%		
		>15 cc	143	27.3%	75.354	0.000
rV40	0.06%–99.52%(6.59%)	≤11%	363	2.5%		
		>11%	138	27.5%	73.846	0.000
aV40	0.04–107.34 cc(4.92 cc)	≤11 cc	409	2.7%		
		>11 cc	92	39.1%	117.321	0.000
rV50	0.00%–98.07%(2.49%)	≤5%	363	1.7%		
		>5%	138	29.7%	92.589	0.000
aV50	0.00–105.78 cc(1.92 cc)	≤4 cc	364	1.6%		
		>4 cc	137	29.9%	93.632	0.000
rV60	0.00%–92.58%(0.25%)	≤2%	422	4.0%		
		>2%	79	38.0%	90.200	0.000
aV60	0.00–99.86 cc(0.20 cc)	≤2 cc	430	4.0%		
		>2 cc	71	42.3%	105.150	0.000
rV66	0.00%–77.40%(0.00%)	≤0.1%	340	0.9%		
		>0.1%	161	27.3%	89.895	0.000
aV66	0.00–83.48 cc(0.00 cc)	≤0.2 cc	361	1.1%		
		>0.2 cc	139	30.2%	101.788	0.000
rV70	0.00%–50.65%(0.00%)	≤0.01%	351	1.7%		
		>0.01%	150	27.3%	81.166	0.000
aV70	0.00–54.64 cc(0.00 cc)	≤0.07 cc	380	2.4%		
		>0.07 cc	121	31.4%	91.021	0.000
Dmax	32.4–84.9 Gy(66.15 Gy)	≤68.85 Gy	321	0.6%	80.616	0.000
		>68.85 Gy	180	25.0%		
Dmean	3.9–35.7 Gy(11.70 Gy)	≤11.55 Gy	273	0.4%	57.347	0.000
		>11.55 Gy	228	20.2%		

**Figure 2:**
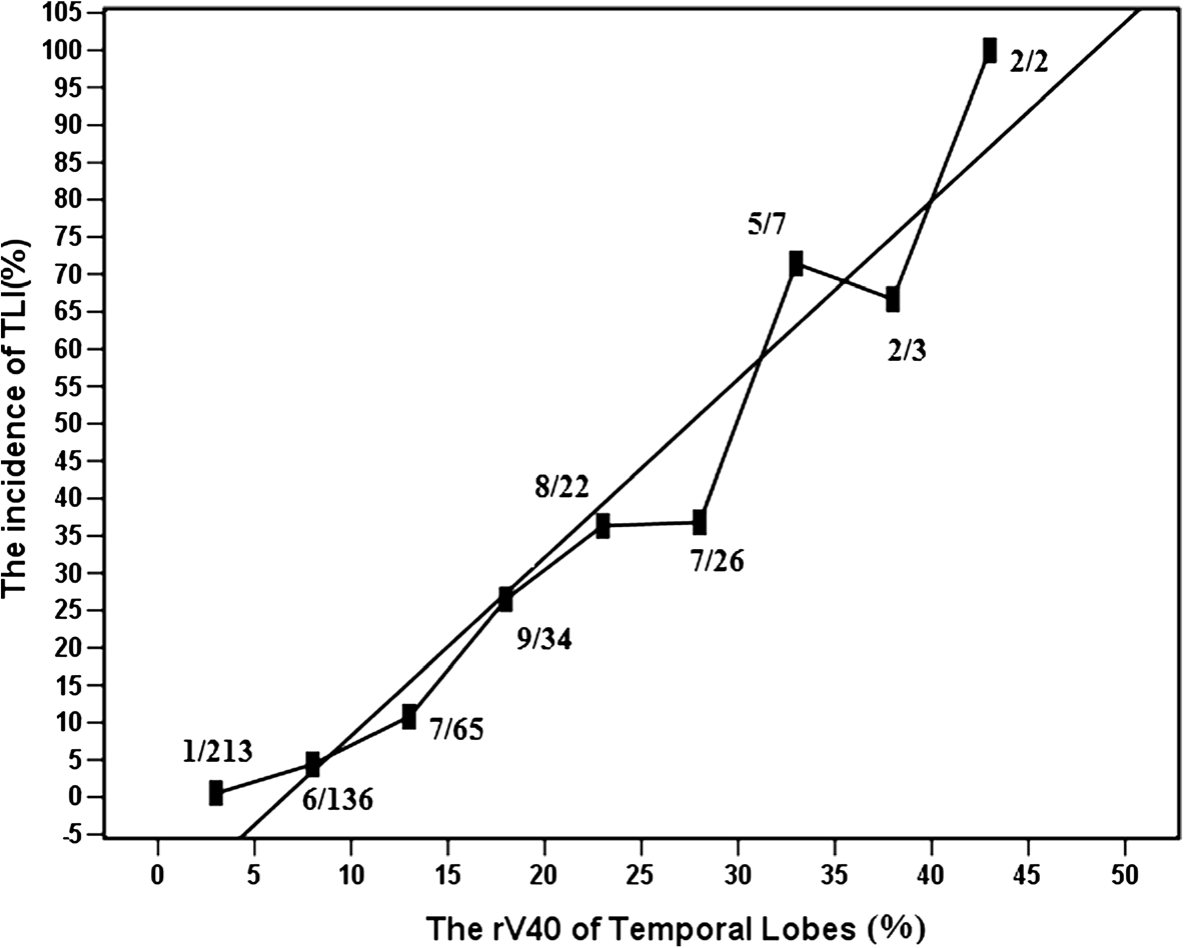
Plot of 5-year incidence as function of rV40 for temporal lobes.

**Figure 3:**
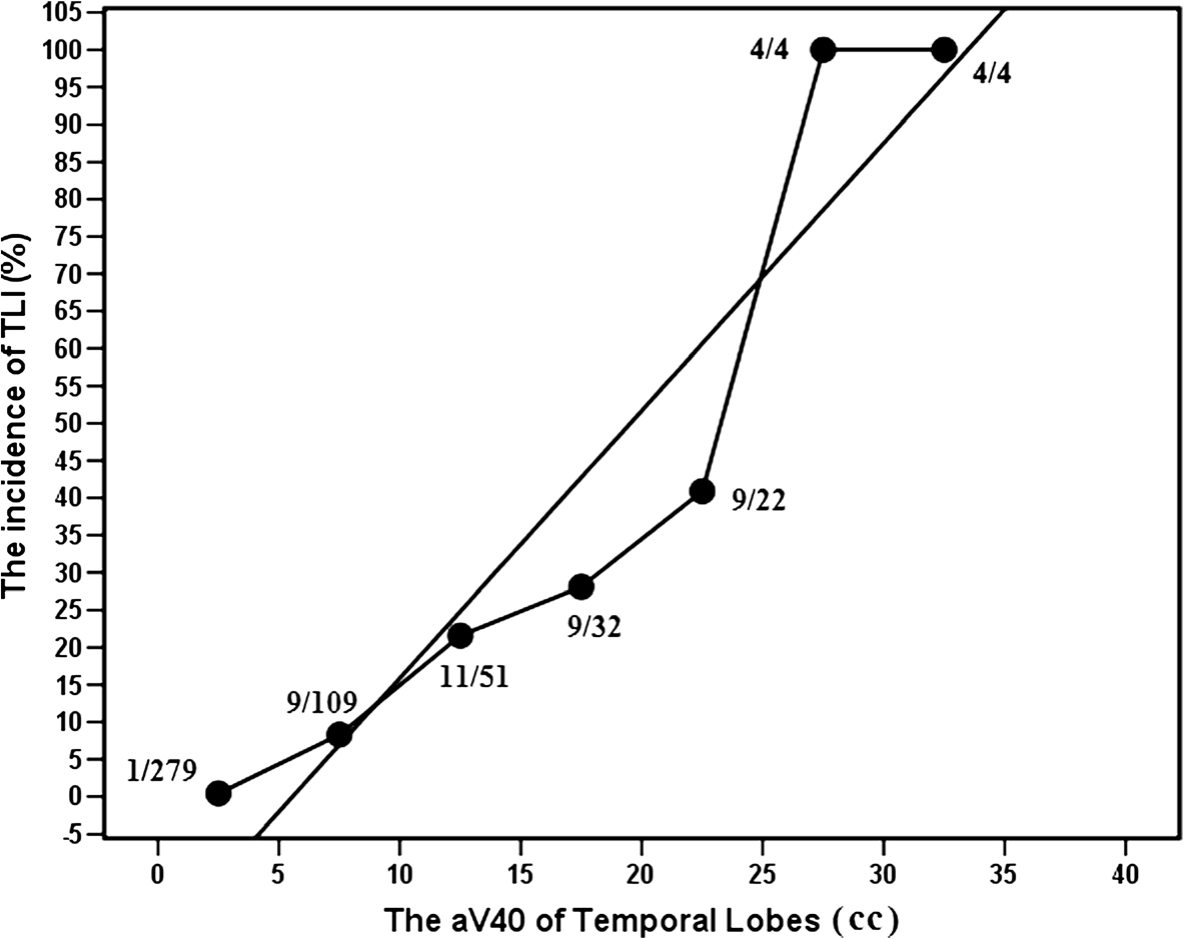
Plot of 5-year incidence as function of aV40 for temporal lobes.

Multivariate analysis by logistic regression showed that rV40 and aV40 were significant factors for TLN. All dosimetric factors investigated in current serials were significantly correlated with the development of TLN, these factors were associated with each other closely and with rV40 and aV40 (Table 2). Because rV40 and aV40 was demonstrated by the multivariate analyses to have a significant influence on TLN, the relationship between rV40 and aV40 and incidence of TLN was plotted in Figure 2 and Figure 3, respectively. The actual 5-year incidence of TLN for 501 TLs were 0.5%, 4.4%, 10.8%, 26.5%, 36.6%, 36.8%, 71.4%, 66.7% and 100% for rV40 < 5%, 5% ≤ rV40<10%, 10% ≤ rV40<15%, 15% ≤ rV40<20%, 20% ≤ rV40<25%, 25% ≤ rV40<30%, 30% ≤ rV40<35%, 34% ≤ rV40<40% and 40% ≤ rV40, respectively (Figure 2). The actual 5-year incidence of TLN were 0.4%, 8.3%, 21.6%, 28.1%, 40.9%, 100.0%, and 100% for aV40 < 5 cc, 5 cc ≤ aV40<10 cc, 10 cc ≤ aV40<15 cc, 15 cc ≤ aV40<20 cc, 20 cc ≤ aV40<25 cc, 25 cc ≤ aV40<30 cc, and 30 cc ≤ aV40, respectively (Figure 3). A linear regression line was fitted to the data points. The slope of the linear regression line were 2.00 ± 0.43% per one percent (95% CI; 1.00 ± 3.02) of rV40 and 3.58 ± 0.59% per cc (95% CI; 2.07 ± 5.10) of aV40 which were significantly different from zero (P <0.01). The incidence of TLN was obviously related to rV40 and aV40.

**Table 2:** Pearson correlation coefficients between dosimetric factors; p < 0.001 in each case

	**rV10**	**aV10**	**rV20**	**aV20**	**rV30**	**aV30**	**rV40**	**aV40**	**rV50**	**aV50**	**rV60**	**aV60**	**rV66**	**aV66**	**rV70**	**aV70**	**Dmax**	**Dmean**
rV10	1.000																	
aV10	0.940	1.000																
rV20	0.940	0.899	1.000															
aV20	0.880	0.946	0.950	1.000														
rV30	0.849	0.829	0.964	0.932	1.000													
aV30	0.796	0.861	0.917	0.968	0.963	1.000												
rV40	0.777	0.773	0.906	0.894	0.973	0.955	1.000											
aV40	0.721	0.784	0.856	0.912	0.932	0.976	0.972	1.000										
rV50	0.690	0.704	0.826	0.836	0.911	0.916	0.975	0.969	1.000									
aV50	0.631	0.695	0.774	0.837	0.865	0.921	0.938	0.979	0.978	1.000								
rV60	0.583	0.619	0.724	0.764	0.819	0.858	0.905	0.932	0.964	0.974	1.000							
aV60	0.521	0.593	0.669	0.746	0.768	0.844	0.859	0.923	0.927	0.973	0.984	1.000						
rV66	0.500	0.554	0.642	0.704	0.738	0.802	0.832	0.886	0.905	0.942	0.978	0.985	1.000					
aV66	0.444	0.525	0.591	0.682	0.691	0.782	0.787	0.869	0.866	0.930	0.951	0.985	0.989	1.000				
rV70	0.466	0.528	0.598	0.670	0.693	0.766	0.789	0.852	0.865	0.911	0.945	0.961	0.987	0.979	1.000			
aV70	0.420	0.506	0.559	0.656	0.658	0.755	0.755	0.843	0.836	0.906	0.926	0.965	0.979	0.992	0.989	1.000		
Dmax	0.681	0.671	0.710	0.683	0.738	0.695	0.712	0.656	0.645	0.582	0.535	0.470	0.430	0.375	0.387	0.343	1.000	
Dmean	0.911	0.887	0.973	0.945	0.975	0.948	0.960	0.927	0.915	0.874	0.839	0.789	0.765	0.717	0.726	0.688	0.726	1.000

## Discussion

It is unavoidable that the lower part of temporal lobe are involved in the radiation fields, and TLN is one of the most serious late complications that may cause focal neurological deficits, memory loss, and other neurocognitive dysfunction after radiotheraphy for NPC [7,15]. In the past decades, 2D-RT was used widely for the control of NPC, and the lower part of temporal lobe was exposed to a large dose of radiation, which resulted in a high incidence of TLN ranges from 0% to 18.6% [4,7,15]. In China, the mean incidence of radiation encephalopathy in NPC patients is 1.9% (range, 0.4%-2.6%) after 2D-RT, and majority of the patients are treated with 2-Gy daily fractions for a total dose of nearly 70 Gy [10]. Although 1ower dose but higher incidence of TLN in Lee series [4,7], this may be mainly due to higher fractional dose. In the Lee et al’s report [7], the 10-year actuarial incidence of TLN was 4.6% for patients irradiated to 60.0 Gy with 2.5 Gy per fraction, and up to 18.6% for those irradiated to 50.4 Gy with 4.2 Gy per fraction. Lee et al. [4,7] revealed that fractional effect is independent significant factor affecting cerebral necrosis. In addition to radiation schedules, different diagnostic criteria and using different radiological examination may also probably contribute to this variation.

In the current study, all TLN happened within 5 years after IMRT. That is to say, there is little probability of occurrence of TLN over 5 years of treatment completion. The incidence of TLN at 5 years in our study was 15.4% (40/259). In Bakst *et al*’*s* prospective trial, 25 NPC patients are treated by IMRT for a total of 70 Gy with 2.34 Gy pre fraction to GTV over a 30-day course, and TLN becomes one of the common late complications with a high incidence at 12% [16]. Comparing with Bakst *et al*’*s* study [16], the incidence of TLN was higher in current study. This might be due to shorter follow-up time in Bakst *et al*’*s* study [16], and more TLN cases are likely to be diagnosed with the follow-up time prolonging. However, it is worthwhile to note that hypofractionation scheme and shorter overall radiation treatment time (OTT) was used in the study by Bakst et al. [16]. Larger fractional dose and shorter overall radiation treatment time will increase the risk of temporal lobe necrosis for NPC patients after RT [4,7]. With IMRT, the incidence of TLN is expected to decrease. Howerver, IMRT seem to be unable to reduce TLN compared with 2D-RT. This may be due to larger fractional dose and shorter OTT used in IMRT. Lawrence *et al*. [11] reported that the brain is especially sensitive to fraction sizes >2 Gy, and Lee *et al*. [4] reported that the OTT had significant impact on the risk of temporal lobe necrosis. To date, the optimal dose and fractionation schemes have not reached a consensus yet. In the first multi-institutional study, the RTOG 0225 trial, 2.12 Gy per fraction is used to treat the primary tumor and the implicated nodes, while 1.8 Gy per fraction is for subclinical disease and elective neck [17].

The precise mechanism that causes TLN remains unknown, but it may be associated with volume and dose of TL irradiated. Lawrence *et al*. [11] have reviewed the published data regarding radiotherapy (RT)-induced brain injury and suggested that incidence and severity is dose and volume dependent. Lawrence *et al*. [11] reporeted that the 5% risk of brain injury at 5 years of the partial brain for conventionally fractionated RT is 72 Gy (range, 60–84). This dose tolerance was concluded from publications concerned 2D-RT for patients with NPC, glioma, or brain metastases, NPC patients were the majority. However, the relationship between TLN and actual dose-volume of irradiated TLs in 2D-RT cannot be analyzed due to the technical limitations. Our experience may be useful for designing IMRT plan for NPC patients.

In the current series, all TLN happened in 40 patients with advanced T-stage. These patients accompanied with the high prevalence of infiltration to the skull base and intracranial tissue or in proximity to them, and a portion of temporal lobe is even delineated as target in some advanced T-stage NPC patients; under this condition, a portion of temporal lobe may be irradiated with higher dose in order to improve target coverage. Concurrent chemoradiotherapy is standard treatment modality for advanced T-stage disease, 36 out of 40 patients (90%) received concurrent chemoradiotherapy in this study. Therefore, it is difficult to determine whether TLI in patients with advanced T-stage is correlated with chemotherapy or higher radiation dose in temporal lobes, and further evidence is needed to resolve this issue.

All dosimetric factors investigated in current serials were significantly associated with the occurrence of TLN by univariate analysis. The dosimetric factors were associated with each other closely (Table 2). In multivariate analysis, rV40 and aV40 were highly predictive of TLN, with incidence of TLN of 2.5% vs. 27.5% in patients having rV40 ≤11% vs. rV40 >11% (p =0.000), and 2.7% vs. 39.1% in patients having aV40 ≤11 cc vs. aV40 >11 cc (p =0.000).

Because rV40 and aV40 were demonstrated by the multivariate analyses to have a significant influence on TLN, and the range of rV40 (0.06%-99.52%) and aV40 (0.04-107.34 cc) were relatively wide, the authors have plotted a dose response curve to determine the probability of TLN as the function of rV40 and aV40. A linear regression has demonstrated a 2.00 ± 0.43% increment of TLN per one percent of rV40 and 3.58 ± 0.59% per cc (95% CI; 2.07 ± 5.10) of aV40. In the current series, the 5-year incidence of TLN for patients with rV40 <10% or aV40 <5 cc is less than 5%. However, the 5-year incidence of TLN in TLs under 15% ≤ rV40 or 10 cc ≤ aV40 is increased significantly and more than 20%. Thus, these results suggest that while IMRT with rV40 <10% or aV40 <5 cc in temporal lobes is relatively safe by using IMRT for NPC patients. Emami’s original estimate for fractionated partial brain RT suggested 5% risk at 5 years for one-third brain irradiated to 60 Gy [9]. Lee *et al*. reported that 64 Gy in 32 fractions with one fraction daily would lead to a 5% necrotic rate in TLs 10 years after 2D-RT in NPC patients [7]. Based on Emami’s [9] and Lee *et al*. [7] reports, 40 Gy might be regard as an odd constraint to use for temporal lobe necrosis. At same time, it seems to be incredulous that 15% ≤ rV40 or 10 cc ≤ aV40 will cause incidence of TLN more than 20%. We consider this may be due to different RT techniques used in different studies. In Emami’s and Lee *et al*’s studies, patients were treated with 2D-RT, though, IMRT was used in our study. Although rV40 and aV40 were the only two factors selected by the multivariate analyses have a significant influence on TLN in current serials. In the study, all dosimetric factors were significantly correlated with the development of TLN, these factors were associated with each other closely and with rV40 and aV40 (Table 2). This correlation could not be observed in 2D-RT for NPC patients. In other words, rV40 and aV40 was correlated with higher-dose volume in IMRT plan. It is well acknowledged that high radiation dose to TLs is relevant to TLN. So it is understandable why rV40 and aV40 might be independent risk factors for the development of TLN in NPC patients treated with IMRT. Due to the correlation among all dosimetric factors, rV40 and aV40 in combination of other dosimetric factors will be better for prediction of TLN for an individual IMRT planning to predict TLN.

## Conclusions

Our findings suggest that rV40 and aV40 were demonstrated by the multivariate analyses to have a significant influence on TLN, and rV40 <10% or aV40 <5 cc in temporal lobes is relatively safe by using IMRT for NPC patients. In the study, all dosimetric factors were significantly correlated with the development of TLN, and these factors were associated closely with each other. Thus, rV40 and aV40 in combination of other dosimetric factors will be better for prediction of TLN. Our experience may be useful for designing IMRT plan using Peacock system for NPC patients, and further evidence is needed to determine whether same conclusion can be drawn in IMRT treatment using modern linear accelerators and treatment planning system. Given few publications of detailed temporal lobe dose–volume and outcomes data of TLN for NPC patients following IMRT with long-term follow-up and the retrospective nature of this report, further evidence will be necessary to confirm relationship between TLN and dosimetric factors.

## Competing interests

There are no financial disclosures or conflicts of interest from any authors.

There are no any actual or potential conflicts of interest exist.

## Authors’ contributions

T-XL designed the study. S-FS, S-MH, FH, YH, C-YC, W-WX, and X-MS collected the data. T-XL, S-FS, S-MH, and FH did the data analysis, interpretation, and wrote the report. T-XL, and S-FS did the statistical analysis. All authors read and approved the final manuscript.

## Funding

Supported by grants from the National Natural Science Foundation of China (No. 30770656).
